# The MiBlend Randomized Trial: Investigating Genetic Polymorphisms in Personalized Responses to Fruit and Vegetable Interventions for Chronic Disease Prevention

**DOI:** 10.3390/antiox14070828

**Published:** 2025-07-04

**Authors:** Julia N. DeBenedictis, Na Xu, Theo M. de Kok, Simone G. van Breda

**Affiliations:** Department of Translational Genomics, Faculty of Health, Medicine & Life Sciences, GROW Institute for Oncology and Reproduction, Maastricht University, P.O. Box 616, 6200 MD Maastricht, The Netherlands

**Keywords:** fruits and vegetables, genetic polymorphisms, human intervention study, bioactive compounds, personalized prevention, chronic disease

## Abstract

Background: The MiBlend Study investigated the effect of consuming different combinations of fruits and vegetables (F&Vs) blends on markers of chronic disease risk and gene expression changes in healthy human subjects. Overall, the increase in F&Vs led to reduced susceptibility to the induction of DNA damage ex vivo, higher antioxidant capacity of plasma, and improved microvasculature as reflected by retinal analysis. As with most dietary intervention studies, inter-individual variability was observed in the responses, which might be the consequence of genetic differences. Therefore, this study aims to identify if genetic variants in relevant genes affect outcomes and responses to the dietary interventions. Methods: The literature review identified 15 polymorphic genes related to phytochemical metabolism, oxidative stress, and detoxification, which were tested in 146 participant samples using TaqMan and PCR analysis. The effect of genotypes on study outcomes was determined via analysis of variance. Results: XRCC1 wildtype carriers were more protected from ex vivo-induced DNA damage after consuming flavanol-rich F&Vs than other variants. XRCC1 is involved in DNA repair, particularly oxidative damage, and its wildtype allele enhances repair efficiency. GSTP1 wildtype carriers had a larger improvement in microvasculature after all F&V blends, especially those rich in polyphenols. GSTP1 polymorphisms likely affect microvascular responses to polyphenol-rich F&V intake by modulating detoxification and fiber-derived butyrate that can influence arterial dilation and endothelial function. Conclusions: Stratifying participants by relevant genetic polymorphisms can reveal predisposed responses to nutrients and guide efforts to personalize disease prevention strategies.

## 1. Introduction

Diet is a major modifier of chronic disease risk, but there is increasing evidence that individuals respond differently to the same diet and, therefore, benefit from more personalized dietary advice [1,2,3]. This heterogeneity in responses to healthful or protective diets, often leading to distinct categories of “responders” and “non-responders,” has introduced ambiguity into the outcomes of nutrition intervention trials [4]. Genetics, among other factors contributing to this diversity, stands out as a critical determinant [5]. Genetic variations can affect the activity and efficacy of proteins responsible for DNA repair, oxidative stress, and the metabolism and detoxification of a wide range of substances, collectively shaping an individual’s personal response to specific dietary compounds [6,7,8,9].

For example, the association between coffee and myocardial infarction has been disputed, with epidemiological studies showing conflicting results [10,11,12]. However, in a study of 2,057,000 Costa Ricans, the participants’ genotype for the caffeine-metabolizing enzyme CYP1A2 was found to play a significant role in whether increased coffee consumption led to an increased risk of myocardial infarction. Of the three possible genotypes, carriers of the homozygous variant genotype, indicative of a “slow-metabolizing” caffeine enzyme, were the only group where an increase in coffee intake led to an increased risk of myocardial infarction. Carriers of the other genotypes, who metabolize caffeine at a quicker rate, did not experience the same deleterious consequences from increased coffee consumption [13]. Overlooking the impact of genetic variability likely contributed to the ambiguity and conflicting evidence found in previous studies.

Intervention studies, such as the one conducted by Wilms et al. 2007 [14], also illustrated the impact of genetic polymorphisms on responses to dietary nutrients. In this study, 168 participants consumed 1 L of blueberry–apple juice for four weeks to assess the potential anti-oxidative and anti-genotoxic effect of the fruit juice. Six genetic polymorphisms were found to significantly influence the outcome of the intervention. Participants bearing Cyp1B1*5 variant alleles benefited more than wildtypes from the intervention’s DNA damage-protecting effects. COMT variants benefited less or even experienced detrimental effects from the intervention when compared to wildtypes. CAT1, NQO1, and GSTT1 genotypes impacted the absorption of ascorbic acid, quercetin, and the antioxidant capacity of plasma, respectively [14]. Studies such as these highlight how genotyping participants in dietary interventions for relevant polymorphisms can enable the identification of response patterns. Stratifying by these genetic groups allows for the creation of targeted recommendations for individuals who would benefit most from specific nutrients.

Similarly, the MiBlend Study was a nutrition intervention study testing the effect of various combinations of fruits and vegetables (F&Vs) on chronic disease risk. The preventative potential of F&Vs is likely in large part due to their phytochemical content. There are various types of phytochemicals of differing chemical structures and therefore categorical classes (e.g., flavonoids, carotenoids, anthocyanins, glucosinolates), and these compounds have been studied for their various roles in chronic disease prevention. Seven different interventions (combinations of F&Vs) were tested in this study. The different combinations (or blends) of F&Vs randomized to each participant were designed to contain an overrepresentation of particular phytochemical classes from common F&Vs, which then increased in complexity. Each participant completed a two-week run-in period to standardize F&V intake to 50 g per day, followed by two two-week intervention phases, with a one-week washout phase in between. Participants were randomly assigned to two out of the seven different blends, and the washout phase was included to prevent any carryover effects, allowing for clear assessment of the impact of increasing F&V intake to 450 g per day during the intervention phases. The results of this study showed an overall increase in the antioxidant capacity of plasma, an improvement in retinal microvasculature phenotype (a marker of cardiovascular disease risk), and the protection of DNA from ex vivo-induced oxidative stress [15]. However, a variation in responses was observed, indicating the relevance of genetic polymorphism analysis. We therefore hypothesize that within our study population, sub-groups exist that, based on genetic predisposition, derive varying degrees of benefit from the protective effects of specific F&V blends. Polymorphisms of interest are those involved in the kinetics of phytochemicals, oxidative stress handling, and phase I and II detoxification enzymes. We chose polymorphisms that significantly affected individual responses from past nutrition studies and that exhibit a sufficiently prevalent frequency to allow for detection in the MiBlend Study population.

## 2. Materials and Methods

### 2.1. Study Population

A total of 146 healthy individuals—comprising 106 women and 40 men between the ages of 18 and 59—were enrolled in this study. Recruitment was conducted through online platforms and printed flyers distributed in Maastricht, The Netherlands. Ethical approval was granted by the Medical Ethics Review Committee of Maastricht University Medical Centre+ (MUMC+) (approval number: NL66118.068.18), and the study was registered with the International Trial Registry Platform (ICTRP) under identifier NTR7556. All procedures complied with the guidelines laid down in the Declaration of Helsinki. Eligible participants were required to be in good health, aged between 18 and 60 years, with a body mass index (BMI) between 18.5 and 27 kg/m^2^. Individuals were excluded if they had a history of alcohol abuse within the past six months, were current smokers or had quit smoking within the last three months, or experienced any gastrointestinal, renal, hepatic, pulmonary, cardiac, endocrine, or metabolic disorders. Additional exclusion criteria included HIV, hepatitis, anemia, current pregnancy, recent use of antibiotics (within three months), regular use of medications (except oral contraceptives), relevant food allergies, adherence to vegetarian or vegan diets, high levels of physical activity (over eight hours per week of vigorous exercise), or concurrent involvement in other intervention trials. A registered Dietitian Nutritionist oversaw participant adherence and monitored dietary compliance throughout the study period.

### 2.2. Study Design

**Figure 1:**
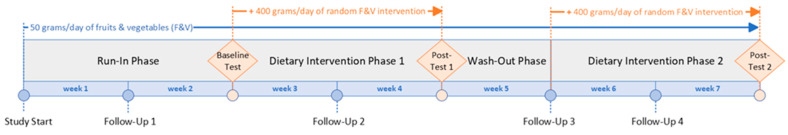
MiBlend study timeline.

The MiBlend study was designed as a randomized controlled crossover trial, aiming to assess how different combinations of fruits and vegetables (F&Vs), along with an overall increase in their intake, influence biomarkers associated with chronic diseases in healthy adults (Figure 1). The study was conducted over a seven-week period. Each participant began with a two-week run-in phase intended to standardize F&V consumption to 50 g per day, ensuring consistent baseline levels across all individuals. Following this, participants underwent a two-week intervention, a one-week washout interval, and a second two-week intervention. During both intervention phases, daily F&V intake was elevated to 450 g to reflect recommended consumption levels and to assess physiological responses within the limited timeframe. Participants were randomly assigned to receive two of seven unique dietary interventions. The inclusion of a one-week washout period between the two interventions was based on prior research [16] and served to minimize any residual effects from the first dietary phase that could influence the outcomes of the second. Sample size calculations and the process of randomization have been described previously [15].

During the entire study duration, participants maintained a baseline intake of 50 g of fruits and vegetables (F&Vs) per day, choosing the types and timing themselves. In the intervention phases, this was supplemented with an additional 400 g of F&Vs, provided as a randomized blend specific to each participant (see Table 1). Participants recorded their daily food intake using the MyFitnessPal app, which was routinely reviewed by a registered Dietitian Nutritionist to ensure adherence. Intake of phytochemical-rich items was regulated throughout the trial. This included two cups of coffee or tea daily. Participants were instructed to avoid dietary supplements, fruit and vegetable juices derived from real produce, red wine, and green tea. Alcohol consumption was allowed only within low-risk thresholds. Specifically, women were restricted to a maximum of two alcoholic beverages per day, up to twice per week, while men were permitted up to three drinks per day, no more than three times per week.

### 2.3. Sample Collections

On both baseline and post-intervention test days, participants reported to the research facility in the morning after an overnight fast. At each visit, a fasting blood sample was collected and participants submitted urine that had been gathered over the preceding 24 h. Additional assessments included measurement of height and weight, as well as a retinal photograph of the right eye. Blood samples were drawn into EDTA-treated tubes and kept on ice until they were processed—either for plasma separation or for comet assay—and subsequently stored at −80 °C. Retinal imaging was performed by a trained technician using a Canon CR-2 non-mydriatic fundus camera (Serial number: S/N 103138) (VITO, Mol, Belgium), and the resulting images were archived for the later analysis of microvascular structures. To maintain consistency, all test-day procedures were carried out in the same sequence at each study visit.

### 2.4. DNA Damage Assessment

The alkaline comet assay was conducted on freshly isolated lymphocytes on the same day that blood samples were collected. Lymphocytes, adjusted to a concentration of 1 × 10^6^ cells/mL, were treated with 25 µM hydrogen peroxide (H_2_O_2_) (Merck, Darmstadt, Germany) and incubated for one hour at 37 °C alongside untreated control samples. Both treated and control cells were embedded in 0.65% low-melting-point agarose (Sigma Aldrich, Steinheim, Germany) and applied in triplicate onto microscope slides. The slides were then placed in a lysis solution (pH 10) composed of 2.5 M NaCl, 100 mM EDTA, 10 mM Tris (Sigma Aldrich, Steinheim, Germany), and 250 mM NaOH (Sigma Aldrich), with 10% DMSO and 1% Triton X-100 (both from Sigma Aldrich, Steinheim, Germany) added freshly before incubation. Lysis was carried out overnight at 4 °C. Slides were then immersed in an electrophoresis buffer (Milli-Q water, 300 mM NaOH, 1 mM EDTA) to allow DNA unwinding for 40 min, followed by electrophoresis at 1 V/cm for 20 min. Afterwards, the slides were rinsed with 1 × PBS and ethanol, dried, and stored in light-protected slide holders at 4 °C. Prior to imaging, the slides were stained with 10 µg/mL ethidium bromide (Cleaver Scientific, Warwickshire, UK), coverslipped, and allowed to incubate for one hour. Imaging was performed using a BIO-TEK Cytation 3 reader equipped with version Gen5 software. Comet images were analyzed using Comet Assay IV software (Version IV, Instem, Stone, UK). For each participant, 50 comets were evaluated, and the average values for % Tail DNA and Tail Moment across the triplicate slides were calculated. To minimize variability, all comet assay procedures and scoring were completed by the same researcher.

### 2.5. Microvasculature Changes

To ensure consistency in image analysis, all fundus photographs from a given participant were evaluated together using MONA-REVA software (version 3.0.0, VITO, Mol, Belgium). The six most prominent arterioles and venules in each image were manually traced, after which the software automatically calculated their calibers and the arteriolar-to-venular ratio (AVR). For each test day, the average values were recorded per participant. To eliminate interrater variability, all vessel diameter assessments were either conducted or verified by the same researcher.

### 2.6. Antioxidant Capacity (TEAC)

Plasma samples collected from participants were thawed after storage at −80 °C prior to analysis. A phosphate-buffered solution was prepared weekly by dissolving sodium phosphate monobasic (Merck, Darmstadt, Germany) in Milli-Q water and adjusting the pH to 7.4 using 1 M NaOH (Merck, Darmstadt, Germany). The radical-generating solution was created by dissolving 2,2′-azinobis-(3-ethylbenzothiazoline-6-sulfonic acid) diammonium salt (ABTS) (Sigma-Aldrich, Steinheim, Germany, CAT: A1888) in phosphate buffer. Separately, 2,2′-azobis-(2-amidinopropane) dihydrochloride (ABAP) (Polysciences, Inc., Warrington, PA, USA, CAT: 08963) was dissolved in phosphate buffer in a beaker. Both solutions were then combined in a flask, followed by the addition of phosphate buffer to reach a total volume of 70 mL. The mixture was incubated in a 70 °C water bath for 10 min, and absorbance readings at 734 nm were taken at 8, 9, and 10 min using an iMark microplate reader (Bio-Rad, Hercules, CA, USA). The target absorbance value was 0.7 ± 0.02. Once this was achieved, the radical solution was cooled on ice and later reheated to 37 °C in a water bath just prior to use. Fresh radical solutions and Trolox standards were prepared daily. The Trolox stock (1 mM) was made by dissolving (±)-6-hydroxy-2,5,7,8-tetramethylchroman-2-carboxylic acid (Trolox) (Sigma-Aldrich, CAT: 238813) in 39.95 mL of phosphate buffer. This stock solution was then serially diluted to create seven calibration standards. Phosphate buffer alone served as the blank and was subtracted from all absorbance values during data processing. For the assay, 280 µL of the prewarmed radical solution (37 °C) was mixed with 15 µL of participant plasma, previously diluted 1:2 in phosphate buffer. The mixture was transferred into a 96-well plate using a multichannel pipette. Absorbance was recorded immediately (t = 0) at 734 nm and again after a 5 min incubation at 37 °C (t = 5). Each sample was measured in triplicate. The change in absorbance between t = 0 and t = 5 for the plasma samples was corrected by subtracting the corresponding change from the blank. A calibration curve was generated from the Trolox standards using their respective absorbance changes, yielding a regression coefficient with an average R^2^ of 0.99. This curve was then used to calculate Trolox equivalent antioxidant capacity (TEAC) values for the plasma samples.

### 2.7. Selection of Gene Polymorphisms

Genetic polymorphisms were selected based on their contribution to the results of similar dietary intervention studies [14,18,19,20,21,22,23,24,25,26,27,28,29,30]. We reviewed human nutrition intervention studies that assessed outcomes related to chronic disease development, such as DNA damage and oxidative stress, or that evaluated the absorption or metabolism of phytochemicals also present in the MiBlend study interventions. These studies were also required to include the examination of single nucleotide polymorphisms (SNPs) or genetic variants in the study population. Genes were selected if they significantly influenced the participants’ outcome response and if the polymorphism’s frequency was greater than 15%, in order to allow for sufficient detection in our study population [31,32]. A total of 15 SNPs were selected for investigation in this study population (Table 2). This includes GSTM1*0, NQO1*2, CAT1*1, GSTT1*0, XRCC1*4, ZBED3, Glu298Asp, COMT, SLC23A1, MTHFR, HNF1A, GSTP1, TCF7L2, BCMO1, and APOC1.

### 2.8. Genotyping Participants

#### 2.8.1. DNA Isolation

DNA extraction was carried out using a Quick-DNA Mini-Prep Kit (Zymo Research, Irvine, CA, USA), following the manufacturer’s instructions with minor modifications. Previously frozen lymphocyte samples stored at −80 °C were thawed on ice. To each cell pellet, 500 µL of Genomic Lysis Buffer was added and mixed by pipetting three times. The samples were then incubated at room temperature for 10 min. Following incubation, the lysate was transferred into a Zymo-Spin™ IICR Column placed in a collection tube and centrifuged at 10,000× *g* for 1 min. The used collection tube was discarded and replaced with a fresh one. Next, 200 µL of DNA pre-wash buffer was applied to the column, followed by centrifugation at the same speed for 1 min. After discarding the flow-through, 500 µL of g-DNA wash buffer was added, and the sample was centrifuged again for 1 min. The column was then moved into a clean 1.5 mL Eppendorf tube, and the lid was left open for approximately 2 min to allow for drying. To elute the DNA, 30 µL of Milli-Q water preheated to 50 °C was added directly onto the column matrix, followed by a 5 min incubation at room temperature and a final centrifugation at maximum speed for 30 s. The concentration and purity of the eluted DNA were assessed via absorbance at 260/280 nm using a NanoDrop Spectrophotometer (Thermo Fisher Scientific, Waltham, MA, USA). Extracted DNA was stored at −20 °C until further analysis.

#### 2.8.2. Genotyping

The participants in the MiBlend study were genotyped for deletion polymorphisms (GSTT1*0 and GSTM1*0) by multiplex PCR. Single nucleotide polymorphisms (NQO1*2, CAT1*1, XRCC1*4, ZBED3, Glu298Asp, COMT, SLC23A1, MTHFR, HNF1A, GSTP1, TCF7L2, BCMO1, and APOC1) were identified by TaqMan assay (Thermo Fisher Scientific, Waltham, MA, USA).

#### 2.8.3. Multiplex PCR Assay

Multiplex PCR was used to analyze polymorphisms in the GSTM1 and GSTT1 genes, with the β-globin gene serving as an internal control. Primer sequences and expected fragment sizes are detailed in Table 3.

Primers, DNA templates, and AmpliTaq Gold^®^ 360 Master Mix (Thermo Fisher Scientific, Breda, The Netherlands) were thawed on ice before use. To prepare the reagent, per 50 samples 5 μL of the primers mentioned above (30 μL in total) were added to 70 μL of nuclease-free Milli Q water. Then 300 μL of Ampli Taq Gold mastermix was added. The mixture was vortexed to make sure the prepared PCR reagent was mixed well. A total of 4 μL of DNA template was added to 8 μL of prepared PCR reagent in a 0.2 mL PCR tube. The PCR tubes were capped and then placed in a thermocycler (Westerburg, The Netherlands). The PCR was carried out with an initial denaturation at 95 °C for 3 min, followed by 40 amplification cycles consisting of 95 °C for 1 min, 56 °C for 1 min, and 72 °C for 1 min, with a final elongation step at 72 °C for 10 min. PCR products were then separated using electrophoresis on a 2% agarose gel.

For gel electrophoresis, a 2% agarose gel was prepared in a 300 mL batch ahead of time and kept warm in a 60 °C oven. From this stock, 20 mL was transferred to a 50 mL beaker, and 2 µL of SYBR Safe DNA gel stain (Thermo Fisher Scientific) was added. The solution was gently mixed until fully homogeneous. The stained gel was then poured into a casting tray with the well comb already in position. Once the gel had completely solidified, it was carefully transferred to the electrophoresis chamber. UltraPure™ TAE buffer (Thermo Fisher Scientific, Waltham, MA, USA) was added to the tank until the gel was fully submerged. The PCR products were mixed with loading dye (Thermo Fisher Scientific, Waltham, MA, USA) and were carefully added into each well of the gel. The electrophoresis apparatus was connected to a PowerPac™ Basic Power Supply (Bio-Rad) and ran at a voltage of 140 V for 20 min. Following electrophoresis, the DNA bands were visualized under UV light by D-DiGit^®^ Gel Scanner (LI-COR, Biotechnology, Homburg, Germany). Compared with DNA ladder (ThermoScientific, Waltham, MA, USA), the PCR product bands were estimated by their sizes to identify the null or the present genotypes of GSTT1 or GSTM1.

#### 2.8.4. TaqMan Assay

The TaqMan SNP gentoyping assay was executed in accordance with the established protocols provided by the manual of TaqMan Genotyping Master Mix (Thermo Fisher Scientific, Waltham, MA, USA). The procedure was initiated by preparing a reaction mixture that included the 5 μL of 2x TaqMan Genotyping Mastermix, 0.5 μL of predesigned SNP probes (Thermo Fisher Scientific, MA, USA), and 4.5 μL of DNA. This 10 μL of mixture was then allocated to each well of a standard 96-well qPCR plate (Bio-Rad, CA, USA).

After preparing all samples, the 96-well plate was sealed tightly with a transparent microplate seal (Greiner Bio-One, Kremsmünster, Austria) to minimize the risk of contamination and prevent evaporation during PCR amplification. The sealed plate was then centrifuged at 2000 rpm for 2 min to ensure proper mixing and to eliminate air bubbles. Subsequently, it was loaded into a CFX Connect Real-Time PCR Detection System (Bio-Rad, CA, USA). Detection settings were configured with Channel 1 for FAM fluorescence and Channel 2 for VIC, enabling the simultaneous detection of two distinct DNA targets within each well.

### 2.9. Statistical Analysis

First, statistical analysis was performed by running a univariate analysis of variance with a Tukey post hoc test in SPSS to determine the effect of gene allele on DNA strand breaks (% Tail DNA and Tail Moment), retinal microvasculature (AVR), and antioxidant capacity (TEAC) for each dietary intervention. The change scores in these outcomes from post-intervention test days compared to baseline were used. Results from Blend 1, Blend 2, and Blend 5 were also combined into one group to form a “polyphenol-rich blends” group, and results from Blend 3 and Blend 6 were combined into one group to form a “carotenoid-rich blends” group to improve the power of our comparisons. *p*-values < 0.05 were considered statistically significant and were expressed as * *p* < 0.05. The false discovery rate was then calculated to derive the adjusted *p*-values after multiple testing (q-values). Significant comparisons with their means, SEM, and *p*- and q-values are reported in Appendix A Table A2. Statistical analyses were performed using IBM Statistics SPSS, version 27 (IBM, Amsterdam, the Netherlands) and Microsoft Excel 2021 (Redmond, WA, USA).

## 3. Results

### 3.1. Participants

**Figure 2:**
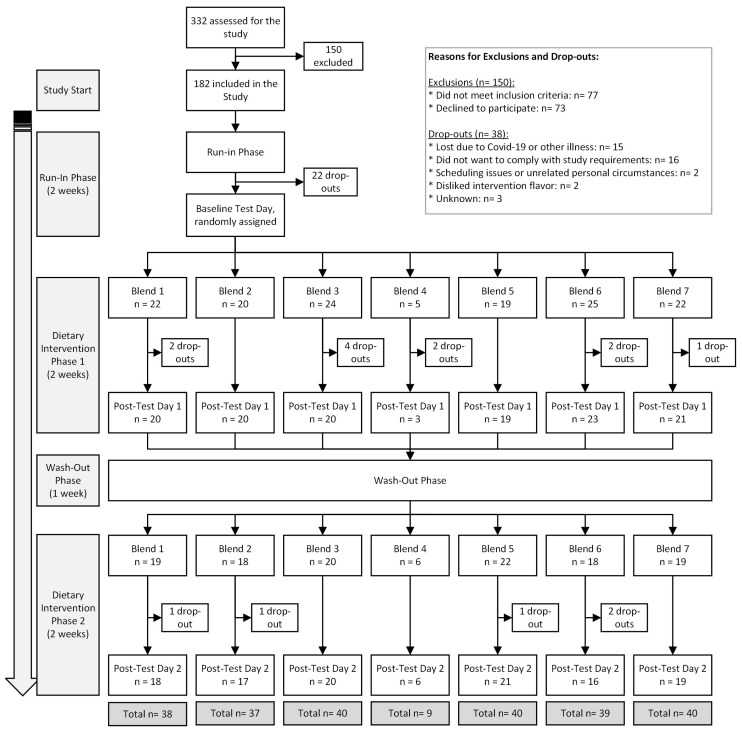
MiBlend study results with number of participants included, excluded, dropped out, and total completed interventions.

A total of 182 participants were initially enrolled in the study, with 146 successfully completing it between March 2019 and May 2022. The trial was concluded once sufficient statistical power was achieved for each intervention group. Most participants dropped out due to illness or challenges in adhering to the study’s guidelines. The flowchart in Figure 2 details the inclusion, exclusion, and dropout distribution of participants. The lower number of completions for Blend 4 was primarily due to participants finding it less tolerable or likable. To minimize unnecessary dropouts, participants who did not tolerate (or anticipated poor compliance) to Blend 4 after an initial tasting were quickly re-randomized to a different blend.

The participants’ sex distribution averaged 27% male and 73% female. The mean age was 27 ± 10 years (ranging from 18 to 59), and the average BMI was 22.7 ± 2.2 kg/m^2^. There were no significant differences in age or BMI between the intervention groups. Although sex distribution differed significantly across groups, this difference was no longer present when the group receiving Blend 4 (which was underpowered) was excluded from the analysis. Table A1 in Appendix A provides a breakdown of age, sex, and BMI distribution.

### 3.2. SNPs

The SNP frequencies within this study population are shown in Table 2 and were in the same range as the frequencies previously reported. This implies that the group of randomly selected participants is representative of the general population. For details of all significant comparisons for AVR, TEAC, and DNA damage, see Appendix A, Table A2.

### 3.3. Effects on DNA Damage

**Figure 3:**
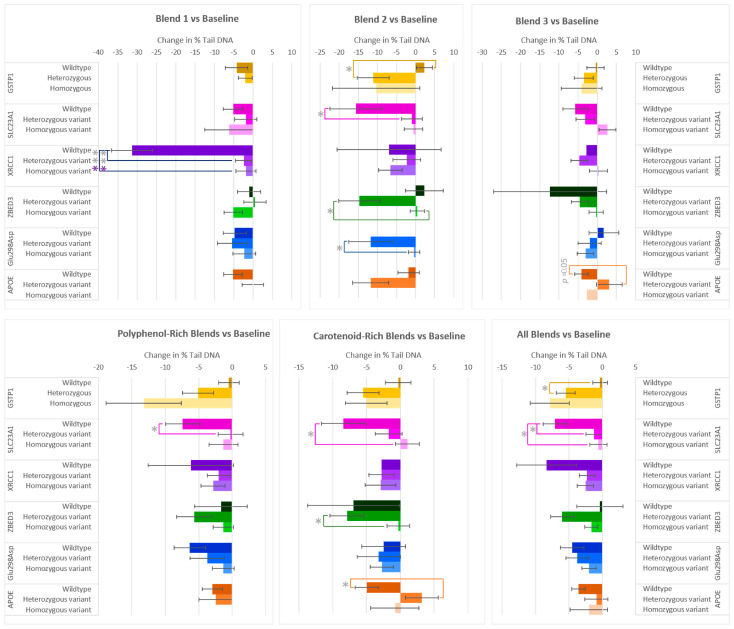
Effect of genotype on changes in % Tail DNA after consuming different combinations of fruits and vegetables for two weeks. The polyphenol-rich blends are a combination of Blends 1, 2, and 5, and the carotenoid-rich blends are a combination of Blends 3 and 6. Data are presented as the change in mean values ± SEM. Absent bars signify an n = 0 for that group, and absent error bars signify an n = 1 for that group. Gray * = the change in the outcome is significantly different for the indicated genotypes within a gene (*p* < 0.05); colored * = the change in the outcome is significantly different for the indicated genotypes within a gene after multiple-testing correction by false discovery rate (q < 0.05). * = *p* < 0.05, *** = *p* < 0.001.

After two weeks of consumption of the various F&V blends, the majority of participants exhibited a reduction in DNA damage (% Tail DNA) (Figure 3). Significant differences in % Tail DNA were found between allele groups from the genes GSTP1, SLC23A1, XRCC1, ZBED3, Glu298Asp, and APOC1. The XRCC1 genotype had a significant effect on the changes in % Tail DNA for flavonoid-rich Blend 1. The wildtype group, although small, exhibited a marked decrease compared to the heterozygous variant and homozygous variant groups. On average, those who had the wildtype allele for GSTP1 did not have a notable reduction in % Tail DNA after the interventions, compared to those with the heterozygous and homozygous variant alleles. This was most pronounced in those who consumed Blend 2 or when considering the effect of all interventions (Blends 1–7), where the reduction in % Tail DNA in the heterozygous group was 13.41% ± 4.82% and 5.13% ± 1.76% lower than the wildtype group, respectively. The SLC23A1 genotype also impacted % Tail DNA responses after the consumption of the F&V blends compared to baseline. On average, wildtype groups showed a larger decrease than heterozygous and homozygous variant groups. After consumption of the anthocyanin-rich Blend 2, the polyphenol-rich blends (Blend 1 + 2 + 5), or all blends, the DNA damage of the wildtype group significantly reduced compared to the heterozygous variant. The % Tail DNA of the SLC23A1 wildtype group was also statistically lower in those in the homozygous group after consumption of the carotenoid-rich blends (Blend 3 + 6) or all blends. In the case of ZBED3 genotypes, the heterozygous variant group had a larger reduction in % Tail DNA than the homozygous variant group after Blend 2 and the carotenoid-rich blends. After two weeks of consuming Blend 2, those with the Glu298Asp heterozygous allele had an 11.40% larger reduction in % Tail DNA than the homozygous variant group. The APOC1 genotype affected the responses in DNA damage after the consumption of Blend 3 and the carotenoid-rich blends, where the wildtype subgroup had a 7.25% ± 3.57 and 8.14% ± 3.23% larger reduction in % Tail DNA than the heterozygous variants.

**Figure 4:**
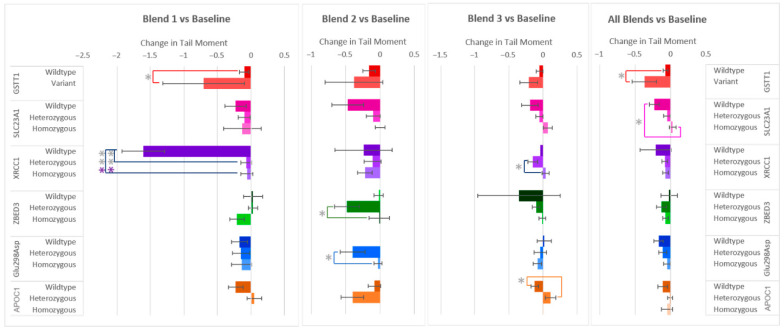
Effect of genotype on changes in Tail Moment after consuming different combinations of fruits and vegetables for two weeks. Data are presented as the change in mean values ± SEM. Absent bars signify an n = 0 for that group, and absent error bars signify an n = 1 for that group. Gray * = the change in the outcome is significantly different for the indicated genotypes within a gene (*p* < 0.05); colored * = the change in the outcome is significantly different for the indicated genotypes within a gene after multiple-testing correction by false discovery rate (q < 0.05). * = *p* < 0.05, *** = *p* < 0.001.

Similarly to the changes in % Tail DNA after the F&V interventions, Tail Moment, the other measure of DNA damage, decreased in most of the samples. Even so, differences in responses among allele groups were also seen (Figure 4). Significant differences in Tail Moment were found between the same allele groups as were found for % Tail DNA (SLC23A1, XRCC1, ZBED3, Glu298Asp, and APOC1), but also in GSTT1 instead of GSTP1. The largest difference in allele group responses was again seen within the gene XRCC1 after the consumption of flavonoid-rich F&V Blend 1, with the wildtype group’s Tail Moment decreasing −1.54 ± 0.27 and −1.55 ± 0.27 more than the heterozygous and homozygous variants, respectively. This finding is consistent with the response in % Tail DNA (Figure 2). The XRCC1 heterozygous group’s Tail Moment was also found to be −0.19 ± 0.05 more reduced than the homozygous group after carotenoid-rich Blend 3. Differences in SLC23A1 allele groups were only found when considering the combined responses of all blends, with wildtype showing again a larger decrease in DNA damage (Tail Moment) compared to the homozygous variant. After consuming the anthocyanin-rich Blend 2, those who have the heterozygous variants of ZBED3 and Glu298Asp again had significant decreases in DNA damage (Tail Moment) compared to the homozygous variant groups. A larger reduction in Tail Moment for the APOC1 wildtype group compared to the heterozygous variant was found after consumption of Blend 3. Finally, the variant group for GSTT1 showed a larger decrease in Tail Moment after consumption of Blend 1 and all blends combined.

### 3.4. Effects on Microvasculature Changes

**Figure 5:**
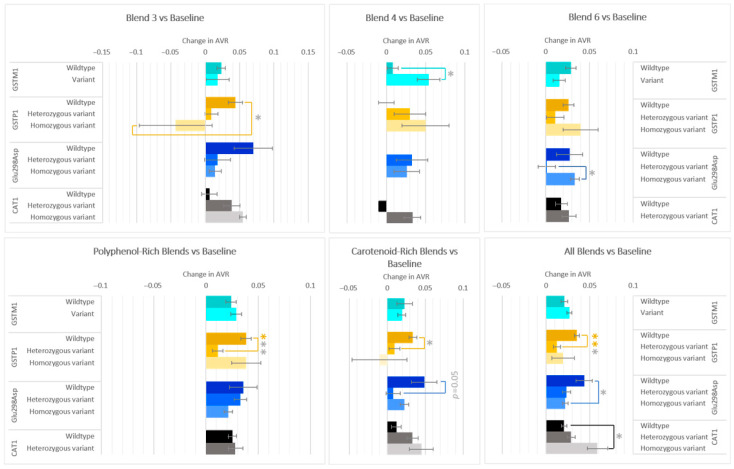
Effect of genotype on changes in AVR after consuming different combinations of fruits and vegetables for two weeks. Data are presented as the change in mean values ± SEM. Absent bars signify an n = 0 for that group, and absent error bars signify an n = 1 for that group. Gray * = the change in the outcome is significantly different for the indicated genotypes within a gene (*p* < 0.05); colored * = the change in the outcome is significantly different for the indicated genotypes within a gene after multiple-testing correction by false discovery rate (q < 0.05). * = *p* < 0.05, *** = *p* < 0.001.

Arteriolar-to-venular (AVR) ratio on average increased after all F&V blends compared to baseline; however, differences in responses differed in allele groups for genes GSTM1, GSTP1, Glu298Asp, and CAT1, and by F&V blend (Figure 5). The variant group of GSTM1 increased 0.046 more than the wildtype group after consumption of glucosinolate-rich Blend 4. Conversely, the GSTP1 wildtype group increased 0.087 ± 0.029 more than the homozygous variant group after carotenoid-rich Blend 3, and increased 0.027 ± 0.007, 0.024 ± 0.01, and 0.023 ± 0.01 more than the heterozygous variant group after interventions were combined for the polyphenol-rich blends (Blend 1 + 2 + 5), carotenoid rich blends (3 + 6), and all blends (1–7), respectively. For Glu298Asp genotype in those who consumed Blend 6, the carotenoid-rich blends, and all blends combined, significant differences were seen between two genotypes. Homozygous variants for CAT1 had a 0.04 ± 0.014 larger increase in AVR than wildtypes when considering all blends combined.

**Figure 6:**
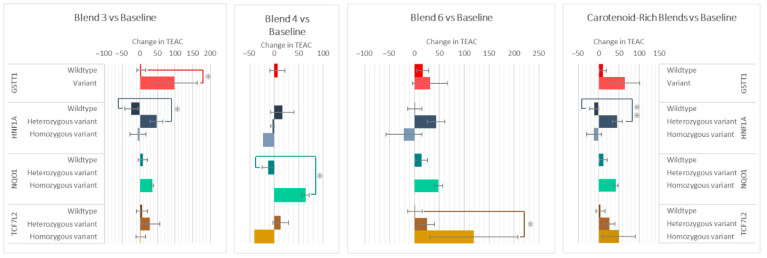
Effect of genotypes on changes in TEAC after consuming different combinations of fruits and vegetables for two weeks. Data are presented as the change in mean values ± SEM. Absent bars signify an n = 0 for that group, and absent error bars signify an n = 1 for that group. Gray * = the change in the outcome is significantly different for the indicated genotypes within a gene (*p* < 0.05). * = *p* < 0.05 and ** = *p* < 0.005.

The genotypes of GSTT1, HNF1A, NQO1, and TCF7L2 impacted the changes in plasma antioxidant capacity (TEAC) after the intake of various F&V blends (Figure 6). Those with the GSTT1 variant had a 95 ± 4 higher increase in TEAC compared to wildtypes after consuming Blend 3. After Blend 3 and the carotenoid-rich blends, the heterozygous variant groups of HNF1A increased 53 ± 35 and 58 ± 25 compared to the homozygous variant groups, respectively. The NQO1 variant group increased 77 ± 22 more than wildtypes after the intake of Blend 4, and the TCF7L2 variant group increased 119 ± 48 after intake of Blend 6.

### 3.5. Effects on Phytochemical Excretion and Absorption

**Figure 7:**
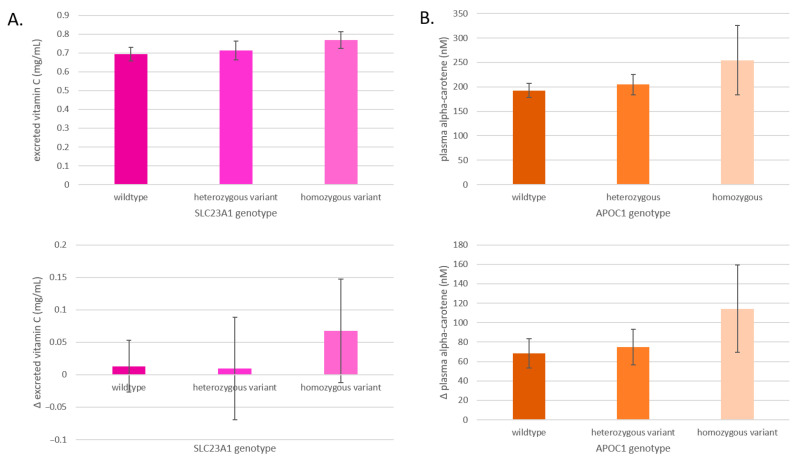
(**A**) Average excreted vitamin C levels by SLC23A1 genotype and changes after all interventions in excreted vitamin C levels by SLC23A1 genotype. (**B**) Average plasma alpha-carotene levels by APOC1 genotype and changes after all interventions in plasma alpha-carotene levels by APOC1 genotype. Data are presented as means ± SEM.

After a 400 g increase in F&Vs, the total concentrations and the change in concentrations of excreted vitamin C and absorbed alpha-carotene differed by SLC23A1 and APOC1 genotype, respectively (Figure 7). The excretion of vitamin C after the F&V interventions increases from SLC23A1 wildtype to the heterozygous variant and to the homozygous variant, and the change from baseline was most pronounced in the homozygous variant group (Figure 7A). The plasma alpha-carotene concentrations after the F&V interventions increase from APOC1 wildtype to the heterozygous variant and to the homozygous variant, and the change from baseline was most pronounced in the homozygous variant (Figure 7B).

## 4. Discussion

In this study, we evaluated the effect of genetic variability on markers of chronic disease risk after the consumption of different combinations of F&Vs, rich in various classes of phytochemicals. The intervention phases amounted to 450 g of F&Vs per day for two weeks, of which 400 g was a specific blend of F&Vs, after two weeks of only 50 g F&Vs intake. With this approach, we can both evaluate and compare the impact of specific blends of F&Vs (or their component phytochemicals) on markers of chronic disease risk, and how an overall increase in F&Vs affects these outcomes by evaluating the changes in these outcomes across all samples. The outcomes of the study include the ability to withstand DNA damage caused by ex vivo-induced oxidative stress (as measured by % Tail DNA and Tail Moment in the comet assay), the total antioxidant capacity of blood plasma (TEAC), and retinal microvasculature changes (AVR) as an indicator of systemic vascular health. The main finding is that inter-individual variation in responses to the F&Vs interventions were partially attributable to genetic variability among the participants. These differences sometimes depend on the type of F&Vs blend that the participants consumed.

The genetic polymorphisms that led to significantly different responses after multiple testing corrections was XRCC1 for DNA damage susceptibility in those who consumed Blend 1, and GSTP1 for microvasculature changes after all F&V blends, especially those rich in polyphenols. The XRCC1 gene encodes a scaffold protein that facilitates the repair of oxidative DNA damage and single-strand breaks by interacting with various DNA repair enzymes. Genetic variations in XRCC1 have been associated with an altered risk of developing cancer [33]. Those with the wildtype allele (although few in number) had an improved ability to withstand oxidative stress compared to those with variant alleles after consuming 400 g of apples and green tea per day for two weeks (Blend 1). These components are abundant in catechins, a specific class of flavonoids that have been reported to reduce DNA damage caused by dimethylbenzanthracene (DMBA) in mice, upregulate DNA repair genes such as XRCC1, and downregulate genes involved in the DNA damage response [34]. In a previous nutrition intervention study where participants consumed 1 L of apple–blueberry juice per day for four weeks, the XRCC1*4 polymorphism was found to be a significant predictor of the intervention’s effect on DNA damage (Tail Moment), with the wildtype also showing the most protective effects [14]. Our results confirm the relevance of this polymorphism and indicate that the interaction between the phytochemicals present in apples and green tea and the XRCC1 gene seem to provide enhanced protection against oxidative stress-induced DNA damage for individuals with the wildtype genotype.

The other gene, GSTP1, belongs to the family of glutathione s-transferase (GST) detoxification enzymes, of which GSTM1 and GSTT1 also belong to, that catalyze the conjugation of potentially harmful compounds to the endogenous antioxidant molecule glutathione [35]. The different classes, such as Mu, Pi, and Theta, have both overlapping and distinct substrate specificities and tissue distributions. Polymorphisms in GST genes may contribute to individual cancer susceptibility and influence individual responses to environmental exposures [36]. In our study, GSTP1 wildtype carriers had a larger improvement in AVR after the increase from 50 to 450 g of F&Vs for two weeks compared to the heterozygous variant. The same difference was not seen between wildtype and homozygous variant carriers, but we suspect this is due to the lower sample size in the homozygous variant group. GSTP1 has been shown in mice to protect against vessel endothelial dysfunction induced by environmental toxins by detoxifying relevant electrophilic compounds [37]. Besides its role in antioxidation and detoxification, GSTP1 has also been shown to be involved in regulating cell-cycle control and cell proliferation in combination with the fiber by-product butyrate, which are both critical pathways for atherogenesis [38]. The interaction between GSTP1 and a non-specific F&V constituent, such as fiber, on arterial dilation and endothelial function supports our findings of polymorphisms in this gene affecting the microvasculature phenotype in a comparison with sufficient power, such as in our combined analyses.

Beyond these findings, attaining statistical power after multiple testing correction was not possible given the number of comparisons required to test 15 genes and 7 interventions and combinations thereof with this study’s sample size. Therefore, the comparisons that have a *p* < 0.05 but a q > 0.05 must be considered as hypothesis generating, since we cannot exclude that some of these statistical differences are false positives.

GSTT1 variant carriers were also found to have a larger reduction in Tail Moment than wildtypes in those who consumed Blend 1 and when combining all F&V interventions. Those with the variant genotype also had higher TEAC, especially after consumption of the carotenoid-rich Blend 3. This finding independently confirms the results of Wilms et al., 2007, whose GSTT1 variant group saw a larger increase in TEAC after consumption of the apple–blueberry juice [14]. The magnitude of change seen in their study is similar to that found in our Blend 6 (Figure 5), which also contains apples and blueberries, but the differences in responses between the GSTT1 genotypes increase after the interventions containing carotenoid-rich F&Vs.

Another gene indicated in affecting resistance to DNA damage after the consumption of F&Vs is SLC23A1, which encodes for a sodium-dependent vitamin C transporter SVCT1. Vitamin C serves as an exogenous antioxidant and necessary enzyme cofactor required for the synthesis of collagen, carnitine, and neurotransmitters [38], and impacts epigenetic modification by stimulating processes like histone demethylation and DNA demethylation [39]. Vitamin C has been extensively investigated for its potential role in the prevention and management of chronic conditions such as cancer and inflammatory bowel disease [40,41,42]. The transporter protein SVCT1 is believed to regulate whole-body vitamin C homeostasis and circulating levels, affecting both absorption by the gastrointestinal tract and mediating reabsorption by the kidneys [43]. In mouse and in vitro models, the global elimination of SLC23A1 was shown to lead to a dramatic decrease in vitamin C concentrations in cells and organs, and nonsynonymous mutations reduced the transporter’s capacity by 90%, respectively [43]. Polymorphisms in SLC23A1 have been shown to predict plasma vitamin C levels and tissue distribution, and are associated with an increased risk of Crohn’s disease [40,42,43]. We expect that polymorphisms in this gene impacted the degree of vitamin C absorbed from F&Vs, particularly that reabsorbed by the kidneys, affecting cellular vitamin C concentrations and how susceptible participants were to the oxidative stress-induced DNA damage. In fact, carriers of the homozygous variant of SLC23A1 excreted a greater amount of vitamin C compared to wildtype and heterozygous carriers after an influx of F&V intake (Figure 7A). Considering our results and those of previous studies, we hypothesize that the greater excretion of vitamin C in those with the gene variants is due to the reabsorption and absorption of vitamin C into cells by their SVCT1 transporter, leading to the reduced capacity to withstand oxidative stress-related insults to their DNA [43,44,45].

The APOC1 gene encodes the apolipoprotein C1, a component of lipoproteins, which is involved in lipid transport and distribution to target tissues. It is primarily expressed in the liver, where it plays a key role in the metabolism of high-density lipoproteins (HDLs) and very low-density lipoproteins (VLDLs) [46]. This SNP is situated about 14 kb from the SNPs that define APOE status, a gene whose variants are well-established for impacting the risk of Alzheimer’s and cardiovascular disease [47]. While direct genotyping of APOE can be challenging due to the high GC content (74%) in its targeted region [48], APOE is often co-inherited with APOC1 [49]. A previous study validated the APOC1 SNP rs4420638 as the best proxy for APOE and the most relevant SNP in modulating Alzheimer’s disease risk [50]. Although the association of the APOC1 genotype with disease risk may partly result from linkage disequilibrium with APOE, independent associations between APOC1 genotype and Alzheimer’s disease have been observed in various ethnic groups [49,51,52]. Thus, the APOC1 genotype may serve as a valuable predictor of APOE status and independently impact LDL cholesterol concentrations [53], C-reactive protein levels (a common inflammation biomarker), and coronary heart disease risk [54]. Carotenoids, lipophilic compounds transported by lipoproteins, are likely influenced by APOC1 gene polymorphisms, impacting their absorption and transport. In our study, individuals consuming carotenoid-rich blends and possessing the wildtype APOC1 allele experienced the greatest reduction in DNA damage compared to those with heterozygous variants. Furthermore, the APOC1 genotype exhibited stepwise differences in plasma carotenoid levels among individuals consuming carotenoid-rich blends (Figure 7B).

In conclusion, our study underscores the crucial role of genetic variability in modulating responses to F&V interventions rich in diverse phytochemical blends, shedding light on personalized prevention strategies for chronic diseases. By assessing markers of chronic disease risk following varied F&V consumption, we revealed that inter-individual differences in responses were partially attributed to genetic variability among participants. Notably, XRCC1 polymorphisms affected DNA damage susceptibility, particularly in response to F&Vs rich in flavonoids such as quercetin and catechins. These findings highlight the relevance of this gene in protecting against oxidative stress-induced DNA damage after the consumption of 400 g per day of apples and green tea for two weeks. Additionally, GSTP1 variants influenced microvasculature changes after all F&V blends, emphasizing the gene’s role in modulating the vascular response after a general increase in F&V intake.

While these findings offer valuable insights, it is crucial to acknowledge the exploratory nature of our study and the limitations inherent in its design. The study’s strengths include its novel approach to evaluating the impact of specific F&V blends on chronic disease risk markers, while also considering genetic variability. The use of a controlled intervention with defined F&V intake and the assessment of multiple relevant biomarkers, such as DNA damage, antioxidant capacity, and vascular health, enhances our understanding of how dietary components interact with genetic factors. However, statistical power was limited by the relatively small sample size, which may have impacted the ability to detect significant differences after multiple testing corrections. Additionally, the exploratory nature of the study means that some findings, while promising, need further validation in larger, more diverse populations. Despite these limitations, the study provides important insights into personalized nutrition and the role of genetic polymorphisms in modulating responses to dietary interventions, laying the groundwork for future research in this evolving field.

## Figures and Tables

**Table 1:** MiBlend dietary interventions. n = number of participants who completed a baseline and post-test for the given blend.

Blend	Composition	Over-Represented Phytochemicals	Fruits and Vegetables (F&V)	n
1	1	Flavonoids (quercetin and catechins)	Apples (400 g) and green tea (2 g in 100 mL water ^a^).	40
2	2	Anthocyanins	Blueberries (100 g), blue grapes (100 g), blackberries (100 g) and raspberries (100 g).	38
3	3	Carotenoids	Tomatoes (133 g), carrots (133 g) and red bell peppers (133 g).	40
4	4	Diallyl sulfide Glucosinulates	Broccoli (133 g), cauliflowers (133 g), and Brussels sprouts (133 g).	10
5	1 + 2	Flavonoids Anthocyanins	Apples (200 g), green tea (1 g in 50 mL water), blueberries (50 g), blue grapes (50 g), blackberries (50 g), amd raspberries (50 g).	41
6	1 + 2 + 3	Flavonoids Anthocyanins Carotenoids	Apples (133 g), green tea (0.66 g in 33 mL water), blueberries (33 g), blue grapes (33 g), blackberries (33 g), raspberries (33 g), tomatoes (44 g), carrots (44 g), and red bell peppers (44 g).	40
7	1 + 2 + 3 + 4 (most complex mixture)	Flavonoids Anthocyanins Carotenoids Diallyl sulfide Glucosinulates	Apples (100 g), green tea (0.5 g in 25 mL water), blueberries (25 g), blue grapes (25 g), blackberries (25 g), raspberries (25 g), tomatoes (33 g), carrots (33 g), red bell peppers (33 g), broccoli (33 g), cauliflowers (33 g), and Brussels sprouts (33 g).	41

^a^ Water was boiled and subsequently cooled for 2 min. Subsequently, 2 g of green tea was added to 100 mL water and steeped for 2 min, after which the green tea was removed [17].

**Table 2:** Identified genetic polymorphisms. Wt = wildtype, Hz = heterozygous, Hm = homozygous. Frequencies of null or deletion alleles are labeled under ‘Hm’.

SNP Name	Full Name	Wildtype	Variation	Amino Acid Change	dbSNP ID	Expected Frequencies (%)			Experimental Frequencies (%)		
						Wt	Hz	Hm	Wt (n)	Hz (n)	Hm (n)
GSTM1*0	Glutathione s-transferase mu 1	Present	Deletion	Deletion	-	51.1	-	48.9	37 (54)	-	63 (92)
NQO1*2	NAD(P)H quinone dehydrogenase 1	GG	G>A/G>C	p.R139W, p.R139G	rs1800566	63	33	4	90.3 (130)	-	9.7 (14)
CAT1*1	Catalase 1	CC	C>G/C>T	N/A	rs1001179	62.7	33	4.3	63.9 (92)	32.6 (47)	3.5 (5)
GSTT1*0	Glutathione S-transferase T1	Present	Deletion	Deletion	-	73	-	27	92.4 (133)	-	7.6 (11)
XRCC1*4	X-ray repair cross-complementing protein 1	TT	T>C/T>G	p.Q399R, p.Q399P	rs25487	11.8	45.1	43	7.6 (11)	48.6 (70)	43.8 (63)
ZBED3	Zinc finger BED domain-containing protein 3	GG	G>A/G>T	N/A	rs4457053	9.2	42.3	48.5	7.6 (11)	32.6 (47)	59.7 (86)
Glu298Asp	Endothelial nitric oxide synthase	TT	T>A/T>G	p.D298E	rs1799983	8.8	41.8	49.4	9.0 (13)	36.1 (52)	54.9 (79)
COMT	Catechol-O-Methyltransferase	GG	G>A	p.V158M	rs4680	26.3	50	23.7	41 (59)	41.7 (60)	17.4 (25)
SLC23A1	Solute carrier family 23 member 1	TT	T>A/T>C/T>G	N/A	rs10063949	38.8	47	14.2	31.9 (46)	41 (59)	27.1 (39)
MTHFR	Methylenetetrahydrofolate reductase	GG	G>A/G>C	p.A263V, p.A263G	rs1801133	44	44.7	11.3	40.3 (58)	44.4 (64)	15.3 (22)
HNF1A	Hepatocyte nuclear factor-1 alpha (HNF-1)	AA	A>C/A>T	p.I27L, p.I27P	rs1169288	45.3	44	10.7	38.9 (56)	51.4 (74)	9.7 (14)
GSTP1	Glutathione S-transferase pi 1	AA	A>G/A>T	p.I105V, p.I105P	rs1695	45	44.2	10.8	47.9 (69)	43.8 (63)	8.3 (12)
TCF7L2	Transcription factor 7-like 2	CC	C>G/C>T	N/A	rs7903146	50.8	40.9	8.2	61.1 (88)	31.9 (46)	6.9 (10)
BCMO1	Beta-carotene 15,15′-monooxygenase 1	CC	C>T	p.A379V	rs7501331	61.7	33.7	4.6	68.1 (98)	28.5 (41)	3.5 (5)
APOC1	Apolipoprotein C1	AA	A>G	N/A	rs4420638	69.1	28	2.8	73.6 (106)	25 (36)	1.4 (2)

**Table 3:** The forward and reverse primer and product size of β-globin, GSTM1*0, and GSTT1*0 for PCR assay. bp = base pair.

Gene	Primer Forward	Primer Reverse	Product Size (bp)
β-globin	5′-CAACTTCATCCACGTTCACC-3′	5′-GAAGAG CCAAGGACAGGTAC-3′	268
GSTM1*0	5′-GAACTCCCTGAAAAGCTAA AGC-3′	5′-GTTGGGCTCAAATATACGGTGG-3′	215
GSTT1*0	5′-TTCCTT ACTGGTCCTCACATCTC-3′	5′-TCACCGGATCATGGCCAGCA-3′	480

## Data Availability

The original contributions presented in this study are included in the article/Appendix A. Further inquiries can be directed to the corresponding author.

## Abbreviations

The following abbreviations are used in this manuscript:

F&Vs	fruits and vegetables
SNP	single nucleotide polymorphism
TEAC	trolox-equivalent antioxidant capacity
Wt	wildtype
Hz	heterozygous
Hm	homozygous
ICTRP	International Trial Registry Platform
AVR	arteriolar-to-venular ratio

## Appendix A

**Clinical Trial Registry:** NL7358 (ICTRP).

**Table A1:** Participant demographics and characteristics. Values by test day are reported in the first three columns. Post-test values by dietary intervention are reported in columns 4–10. Values reported are the estimated means after statistical analysis with the standard error.

	Baseline	Post-Test 1	Post-Test 2	Blend 1	Blend 2	Blend 3	Blend 4	Blend 5	Blend 6	Blend 7
N	146	126	117	38	37	40	9	40	39	40
% Female	73%	74%	72%	68%	79%	60%	40%	78%	75%	88%
Age (yr)	27 ± 10	27 ± 10	27 ± 9	27 ± 9	26 ± 9	27 ± 11	34 ± 13	27 ± 10	25 ± 7	28 ± 9
BMI (kg/m2)	22.7 ± 2.2	22.6 ± 2.2	22.7 ± 2.2	22.6 ± 2.2	22.1 ± 2.5	22.1 ± 2.0	23.3 ± 2.6	22.5 ± 2.1	22.9 ± 2.4	22.6 ± 2.2

**Table A2:** All significant within-gene comparisons for AVR (arteriolar-venular ratio), Tail DNA, Tail Moment, TEAC (Trolox-equivalent antioxidant capacity). The change in mean represents the change in mean from baseline after the genotype group’s two-week intervention. Data is presented for comparisons where *p* < 0.05. *p*-values after false discovery rate correction are reported as q-values.

Outcome	Intervention Group	Genotype		Δ Mean	SEM	n	*p*-Value	q-Value
AVR	Blend 3	GSTP1	Wt	0.04	0.039	18	0.012	0.444
			Hm	−0.04	0.092	3		
	Blend 4	GSTM1	Wt	0.01	0.016	5	0.021	0.42
			V	0.05	0.032	5		
	Blend 6	Glu298Asp	Hz	0	0.038	14	0.009	0.33
			Hm	0.03	0.022	21		
	Flavonoid-rich (Blend 1 + 2 + 5)	GSTP1	Hz	0.01	0.032	47	0	0.01
			Wt	0.04	0.038	63		
	Carotenoid-rich (Blend 3 + 6)	GSTP1	Hz	0.01	0.041	33	0.035	1.365
			Wt	0.03	0.034	41		
	All blends	CAT1	Wt	0.02	0.038	152	0.019	0.371
			Hm	0.06	0.033	8		
		Glu298Asp	Wt	0.04	0.046	24	0.033	0.429
			Hm	0.02	0.034	130		
		GSTP1	Hz	0.01	0.036	101	5 × 10^−5^	0.002
			Wt	0.04	0.037	124		
Tail DNA	Blend 1	XRCC1	Wt	−31.28	7.531	2	0.001	0.026
			Hm	−1.86	9.907	15		
		XRCC1	Wt	−31.28	7.531	2	0.001	0.014
			Hz	−2.42	9.222	19		
	Blend 2	ZBED3	Hm	0.46	8.218	19	0.009	0.279
			Hz	−14.8	18.657	12		
		Glu298Asp	Hz	−11.8	20.556	13	0.025	0.258
			Hm	−0.4	6.918	21		
		SLC23A1	Wt	−15.64	20.564	9	0.036	0.279
			Hz	−1	11.007	16		
		GSTP1	Hz	−11.14	16.351	15	0.024	0.372
			Wt	2.27	8.344	16		
	Flavonoid-rich (Blend 1 + 2 + 5)	SLC23A1	Wt	−7.4	15.142	35	0.044	1.54
			Hz	−0.26	12.696	47		
	Carotenoid-rich (Blend 3 + 6)	ZBED3	Hm	−0.3	11.076	44	0.041	0.478
			Hz	−7.9	10.945	19		
		SLC23A1	Wt	−8.45	14.137	19	0.028	0.98
			Hm	1.04	7.733	19		
		APOE	Hz	3.15	9.67	16	0.037	0.648
			Wt	−4.98	11.788	48		
	All blends	SLC23A1	Wt	−7.09	14.665	67	0.008	0.31
			Hz	−1.21	11.938	97		
		SLC23A1	Wt	−7.09	14.665	67	0.014	0.18
			Hm	−0.59	9.141	51		
		GSTP1	Wt	−0.34	11.748	115	0.011	0.21
			Hz	−5.47	13.162	85		
Tail Moment	Blend 1	XRCC1	Wt	−1.61	0.453	2	8 × 10^−6^	1 × 10^−4^
			Hm	−0.06	0.36	15		
		XRCC1	Wt	−1.61	0.453	2	6 × 10^−6^	2 × 10^−4^
			Hz	−0.07	0.36	19		
		GSTT1	Wt	−0.1	0.418	33	0.042	0.434
			V	−0.71	1.057	3		
	Blend 2	ZBED3	Hm	−0.01	0.292	19	0.019	0.589
			Hz	−0.48	0.628	12		
		Glu298Asp	Hz	−0.4	0.659	13	0.026	0.401
			Hm	−0.03	0.261	21		
	Blend 3	XRCC1	Hz	−0.15	0.306	18	0.046	0.805
			Hm	0.04	0.215	17		
		APOE	Hz	0.11	0.237	9	0.035	1.225
			Wt	−0.12	0.272	26		
	All blends	GSTT1	Wt	−0.07	0.475	204	0.042	0.82
			V	−0.37	0.565	11		
		SLC23A1	Wt	−0.23	0.568	67	0.012	0.47
			Hm	0.03	0.353	51		
TEAC	Blend 3	GSTT1	Wt	3.97	75.001	37	0.048	0.94
			V	99	111.879	3		
		HNF1A	Hz	47.67	77.536	18	0.023	0.9
			Wt	−23.81	77.529	16		
	Blend 4	NQO1	Wt	−12.5	29.569	6	0.014	0.266
			Hm	64.5	10.607	2		
	Blend 6	TCF7L2	Wt	0.95	64.998	20	0.049	1.81
			Hm	119.5	125.158	2		
	Carotenoid-rich (Blend 3 + 6)	HNF1A	Hz	45.74	73.418	35	0.003	0.117
			Wt	−11.45	68.646	33		
